# Atypical Antipsychotics and the Human Skeletal Muscle Lipidome

**DOI:** 10.3390/metabo8040064

**Published:** 2018-10-13

**Authors:** Kyle J. Burghardt, Kristen M. Ward, Elani J. Sanders, Bradley H. Howlett, Berhane Seyoum, Zhengping Yi

**Affiliations:** 1 Department of Pharmacy Practice, Eugene Applebaum College of Pharmacy and Health Sciences, Wayne State University, Detroit, MI 48202, USA; gl7527@wayne.edu; 2 Department of Clinical Pharmacy, College of Pharmacy, University of Michigan, Ann Arbor, MI 48109, USA; kmwiese@med.umich.edu (K.M.W.); esand@med.umich.edu (E.J.S.); 3 Division of Endocrinology, School of Medicine, Eugene Applebaum College of Pharmacy and Health Sciences, Wayne State University, Detroit, MI 48202, USA; bseyoum@med.wayne.edu; 4 Department of Pharmaceutical Science, Wayne State University, Detroit, MI 48202, USA; zhengping.yi@wayne.edu

**Keywords:** lipidomic, antipsychotic, mood stabilizer, muscle

## Abstract

Atypical antipsychotics (AAPs) are a class of medications associated with significant metabolic side effects, including insulin resistance. The aim of this study was to analyze the skeletal muscle lipidome of patients on AAPs, compared to mood stabilizers, to further understand the molecular changes underlying AAP treatment and side effects. Bipolar patients on AAPs or mood stabilizers underwent a fasting muscle biopsy and assessment of insulin sensitivity. A lipidomic analysis of total fatty acids (TFAs), phosphatidylcholines (PCs) and ceramides (CERs) was performed on the muscle biopsies, then lipid species were compared between treatment groups, and correlation analyses were performed with insulin sensitivity. TFAs and PCs were decreased and CERs were increased in the AAP group relative to those in the mood stabilizer group (FDR *q*-value <0.05). A larger number of TFAs and PCs were positively correlated with insulin sensitivity in the AAP group compared to those in the mood stabilizer group. In contrast, a larger number of CERs were negatively correlated with insulin sensitivity in the AAP group compared to that in the mood stabilizer group. The findings here suggest that AAPs are associated with changes in the lipid profiles of human skeletal muscle when compared to mood stabilizers and that these changes correlate with insulin sensitivity.

## 1. Introduction

Bipolar Disorder is a severe mental illness estimated to affect approximately 3% of the United States adults each year and carries with it significant social, economic and disability burden [1,2,3,4]. Treatment of bipolar disorder includes both non-pharmacologic and pharmacologic strategies. The pharmacological strategies consist of various medication classes including mood stabilizers and antipsychotics. Despite the efficacy, approximately 50% of bipolar disorder patients become non-adherent on long-term treatment [5,6,7]. Although the reasons for non-adherence are varied, a major contributor is its metabolic side effects. The atypical antipsychotics (AAPs) carry significant risk for metabolic side effects, including weight gain, insulin resistance, and metabolic syndrome, which is thought to be partly responsible for non-adherence [8]. In addition to non-adherence, the metabolic side effects of AAPs contribute to the elevated rates of cardiovascular mortality within the severely mentally ill when compared to the general population [9,10]. In contrast, the class of mood stabilizers, such as lithium and anticonvulsants, has lower metabolic risk compared to AAPs [11]. Thus, there is a clear need to understand the mechanisms of AAP-associated metabolic side effects to decrease non-adherence, morbidity, and mortality within populations that need their therapeutic benefits.

The molecular mechanisms underlying AAP-associated metabolic side effects are likely multifactorial involving several biological pathways and processes [12,13,14]. To date, most human studies have investigated various “omic” features secondary to AAPs using clinically accessible peripheral blood samples (i.e., plasma or serum) [15,16,17,18,19,20,21]. Although these studies provide important evidence into potential mechanisms, they may not capture the tissue-specific effects of AAPs and their relation to metabolic side effects. This is especially important with AAPs when considering their large volume of distributions, which may have multisystem implications [22]. The skeletal muscle is the main tissue involved in insulin-stimulated glucose uptake, and its dysfunction is thought to be a primary contributor to insulin resistance [23,24]. Despite the known importance of skeletal muscle in the development of insulin resistance, this tissue has not been thoroughly investigated in humans for its role in AAP-induced insulin resistance. However, work has previously identified possible changes in skeletal muscle gene and protein regulation with AAP treatment and insulin resistance in human muscle and pre-clinical cell models of muscle [25,26]. Further investigation of changes in downstream gene products, such as the metabolome, is needed to understand the functional molecular effects of AAPs on skeletal muscle.

Lipidomics, a subfield of metabolomics, is the study of the complete set of lipids found within a sample or specimen. Given the multiple critical roles that lipids play in cellular mechanisms, the lipidome may aid in understanding the efficacy and side effects of AAP treatment. To this end, several studies have investigated lipidomic changes in peripheral blood samples from various clinical populations treated with AAPs [18,19,27,28,29,30,31]. These studies have demonstrated that AAPs can influence the peripheral lipidome of several lipid species, which may be correlated to treatment efficacy or side effects. Additionally, work has identified lipidomic changes in the brain of post-mortem samples from patients who were on AAPs compared to healthy control samples [32]. This work serves to link the peripheral findings to a tissue responsible for the therapeutic benefits of AAPs. To date, lipidomic work in the periphery has not been correlated to specific tissues that may play a potential role in AAP side effects.

Within the current study, we aimed to analyze lipidomic features of human skeletal muscle to understand the potential molecular changes that may play a role in AAP-associated insulin resistance. This was done by analyzing total fatty acids (TFAs), phosphatidylcholines (PCs) and ceramides (CERs) from muscle biopsies obtained from bipolar disorder patients on maintenance of AAP therapy compared to an active treatment control group of patients on mood stabilizer therapy. The overall hypotheses were that AAP treatment would be associated with significant shifts in skeletal muscle lipidomic features when compared to mood stabilizer treatment and that these shifts would be correlated to insulin sensitivity.

## 2. Results

### 2.1. Patient Population

The biopsied skeletal muscle was obtained from 30 participants and included in the lipidomic analyses. The average age of the included participants was 43.5 ± 14.3 years and 60% were female. Sixty percent of participants were self-categorized as Caucasian with 33% as African-American. Half of the participants were currently on an AAP and the other half were on a mood stabilizer. AAPs included quetiapine (50%), risperidone (25%), olanzapine (12.5%) and lurasidone (12.5%), and mood stabilizers included lamotrigine (40%), lithium (33%) and valproic acid (27%). Overall, the population had an average body mass index (BMI) of 32.3 ± 8.3 m/kg^2^ and an Insulin Sensitivity Index (ISI) of 10.8 but had peripheral lipid levels within normal limits. Table 1 contains summarized demographic and clinical data of the included participants by treatment with an AAP or a mood stabilizer. Of note, the AAP group was not significantly different compared to the mood stabilizer group, except in ISI (*p* = 0.0220).

Due to the critical importance of accounting for sex differences in metabolic “omic” studies like metabolomics and lipidomics, we included baseline analyses that compared skeletal muscle lipidomic profiles based on sex [33,34,35,36,37,38]. This analysis did not identify any significant differences in individual lipid species between males and females, suggesting no baseline differences due to sex within this specific study (data presented in Table S1, Supplementary Materials).

### 2.2. Lipidomic Analyses of Skeletal Muscle

**Figure 1:**
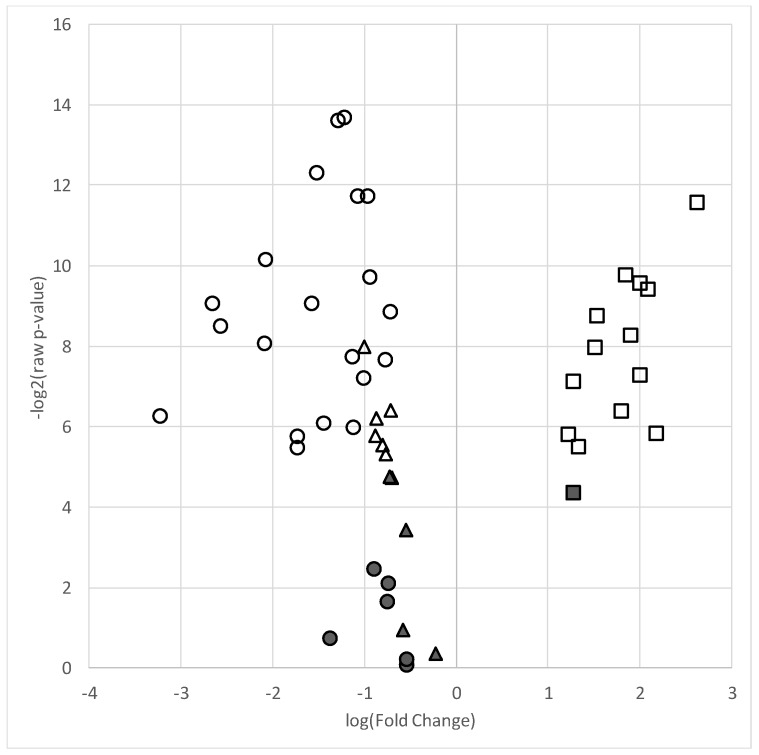
Volcano plot of lipid species in the AAP-treated group relative to the mood stabilizer group. Plot depicts log of –log2 *p*-value (y-axis) versus log base 2 of fold change (x-axis) of each individual lipid included in the analyses. Symbols higher on the y-axis indicate a more significant *p*-value and the greater distance from the center (“0”) indicates a decrease (negative log fold change) or increase (positive fold change) in the lipid level of the AAP group relative to the mood stabilizer group. ● = total fatty acids, ▲ = phosphatidylcholines, ■ = ceramides. Filled shapes are non-significant and empty (non-filled) shapes are significant based on a false discovery (*q*-value < 0.05).

A total of 26 skeletal muscle TFAs, 11 PCs and 14 CERs (51 total lipids) met detection criteria (present in ≥75% of samples) and were included in analyses. A total of 20 TFAs and 6 PCs were decreased in the AAP group relative to those in the mood stabilizer group (all FDR *q*-values < 0.05 and 0.66 fold change). Within the CERs, all but one species was significantly increased in the AAP group relative to those in the mood stabilizer group (fold change > 1.5). Table 2 contains lipid species fold changes, raw *p*-values, and FDR *q*-values. Figure 1 represents a volcano plot of the lipid differences between the AAP and mood stabilizer groups.

### 2.3. Skeletal Muscle Lipidomic Correlation to Insulin Sensitivity

**Figure 2:**
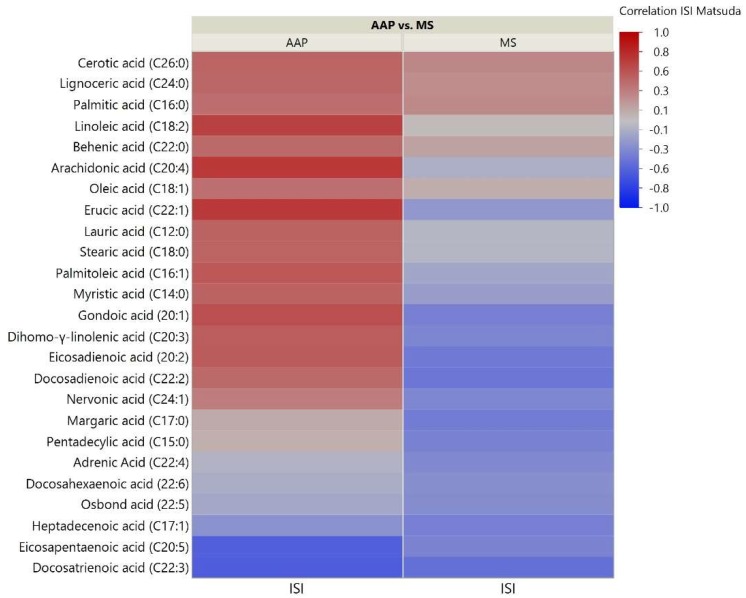
Correlation heatmap of insulin sensitivity and TFAs. The heatmap shows degree of correlation between individual TFA species and insulin sensitivity measured by the Matsuda Insulin Sensitivity Index (ISI). Lipids are ordered by the average ascending correlation value. AAP: atypical antipsychotic, and MS: mood stabilizer.

**Figure 3:**
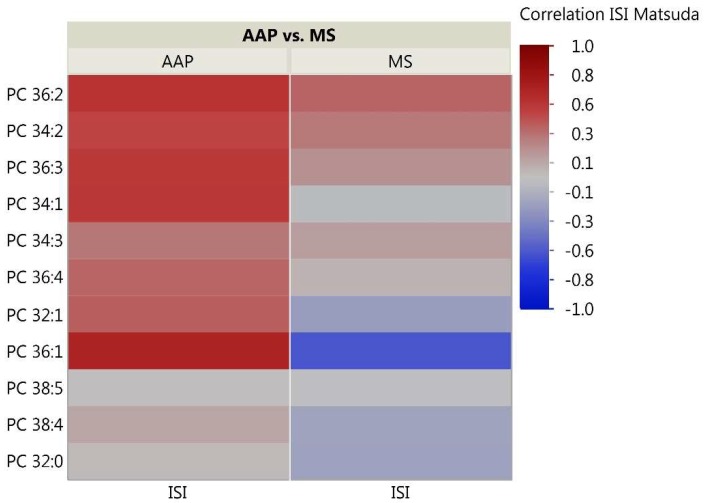
Correlation heatmap of insulin sensitivity and PC Lipids. The heatmap shows degree of correlation between individual PC species and insulin sensitivity measured by the Matsuda ISI. Lipids are ordered by the average ascending correlation value. AAP: atypical antipsychotic, and MS: mood stabilizer.

**Figure 4:**
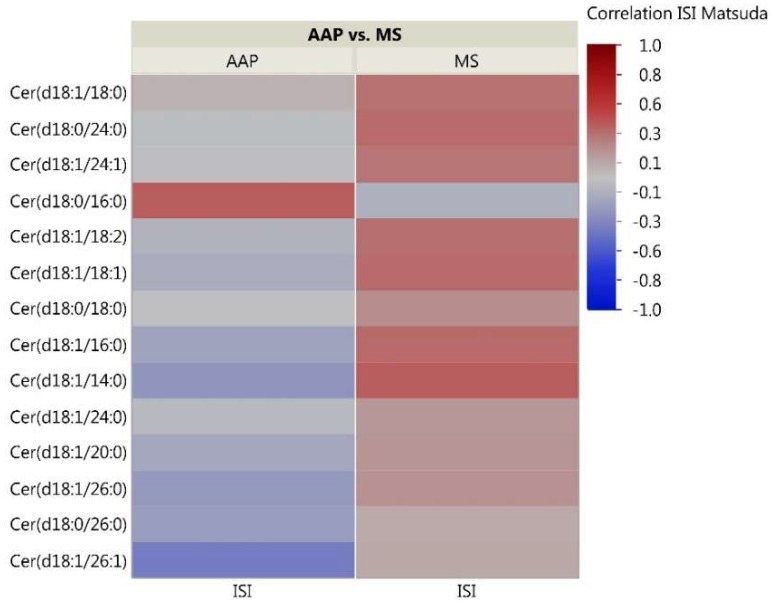
Correlation heatmap of insulin sensitivity and CER lipids. The heatmap shows degree of correlation between individual CER species and insulin sensitivity measured by the Matsuda ISI. Lipids are ordered by the average ascending correlation value. AAP: atypical antipsychotic, and MS: mood stabilizer.

As the skeletal muscle is the primary tissue for glucose uptake and therefore plays a critical role in the pathogenesis of insulin resistance, we analyzed correlations between skeletal muscle lipids and insulin sensitivity measured through the Matsuda ISI. This formula is a “dynamic” assessment of insulin sensitivity that may provide a better reflection of whole-body insulin sensitivity when compared to the gold-standard hyperinsulinemic euglycemic clamp [39]. Correlation analyses for the 51 investigated lipids with insulin sensitivity identified some distinct patterns between the AAP and mood stabilizer groups (Figure 2, Figure 3 and Figure 4). The number of positive relationships was larger in the AAP group for TFAs (20 positive relationships) and PCs (9 positive relationships) compared to that in the mood stabilizer group (5 and 6 positive relationships in TFAs and PCs, respectively). CERs exhibited more negative relationships with ISI in the AAP group (8 negative relationships) compared to those in the mood stabilizer group (1 negative relationship). Several individual lipids showed opposing relationships in the AAP and mood stabilizer groups. For example, opposing relationships were observed for Arachidonic acid, Myristic acid, Docosenoic acid, PC34:1, PC36:1, Cer(d18:1/18:1), Cer(d18:1/16:0) and Cer(d18:1/26:0). Overall, the correlations for CERs and insulin sensitivity were smaller (0.4 and less). *p*-value heatmaps are presented in Supplementary Materials.

## 3. Discussion

Within this study, we investigated the skeletal muscle TFA, PC and CER lipid composition of patients with bipolar disorder on either a maintenance regimen of AAPs or mood stabilizers. This is the first investigation of human skeletal muscle lipidomic features within this population. We identified several differences in lipid features as well as some opposing correlations with insulin sensitivity between groups. The findings here suggest that psychopharmacologic treatment may influence the underlying molecular features of the skeletal muscle, which may contribute to treatment side effects.

### 3.1. Atypical Antipsychotics May Cause Lipid Specific Changes in the Human Skeletal Muscle

Several TFAs were decreased in the skeletal muscle of AAP-treated subjects relative to those in mood stabilizer subjects. These TFAs are involved in various pathways including fatty acid synthesis, elongation, and metabolism. In vivo studies in rodent models have demonstrated that some antipsychotics, but not all, increase the synthesis of peripheral and central nervous system polyunsaturated fatty acids [40]. This is supported by several peripheral blood lipidomic studies within humans that suggest antipsychotics alter, and often increase, most fatty acid species after treatment ranging from four to 12 weeks [18,19,30,41]. In contrast, Albaugh and colleagues demonstrated that short-term treatment with olanzapine, an AAP associated with a higher rate of insulin resistance and weight gain caused a reduction in free fatty acids within *gastrocnemius* muscle tissue, which may be supported by our findings here [42]. Specifically, they suggest that there is a decrease in body-wide lipolysis, along with increased fatty acid tissue deposition that may explain this paradoxical identification of decreased muscle free fatty acids after AAP treatment [18,43,44]. Within their study, Albaugh and colleagues identified decreased muscle malonyl-CoA, a derivative of the dicarboxylic acid malonic acid, suggesting higher rates of fatty acid oxidation. These changes in lipid composition can occur independently of weight gain, which may assist in explaining the direct insulin resistance caused by AAPs [45]. Our work partially supports these findings with decreased skeletal muscle TFA levels in bipolar patients treated for an average of 7.2 months with an AAP when compared to similar patients on mood stabilizers. Although our study did not measure malonyl-CoA, decreased amounts lead to increased fatty acid oxidation and decreased fatty acid synthesis and elongation. This hypothesis may help to explain the lower amounts of skeletal muscle TFAs in AAP-treated patients relative to those in mood stabilizer patients, while the majority of work in the peripheral blood has identified increases in various fatty acid species. Future work will be needed to correlate these preclinical findings with the findings from this study by assessing skeletal muscle fatty acids in prospectively treated patients to understand the short- and long-term effects of AAPs on skeletal muscle.

Our study also identified lower PC lipids in the skeletal muscle of AAP-treated patients compared to mood stabilizer patients. Previous work has demonstrated that antipsychotics may have effects on peripheral blood and brain PC levels in humans [16,18,19,30,32,46]. Findings from these studies suggest that AAPs may increase peripheral PC levels following treatment compared to healthy controls, psychiatric controls or first-episode AAP- naïve patients. However, this may be PC species- and patient-dependent since some PC species were decreased after AAP treatment of schizophrenia patients with recurrent episodes [19]. Others have demonstrated that AAP treatment was associated with a decrease in PCs within the grey matter of AAP-treated schizophrenia patients compared to controls [32]. Together these findings, along with our results presented here, may suggest that the effects of AAPs are tissue-specific and that peripheral changes in PCs may not correlate with other tissues in the body. Future work in both clinical and preclinical models are needed to understand if the effects of AAPs are tissue-dependent and the interaction, if any, between the peripheral and skeletal muscle lipidomes.

In contrast to our analyses in TFAs and PCs, CER species were elevated in the AAP group relative to those in the mood stabilizer group. CER, lipids within the sphingolipid class, play a role in psychiatric disease due to their important function in cell bilayers and cell signaling [47]. Despite work suggesting that CER metabolism may be disrupted in antipsychotic-naïve schizophrenia subjects, work has not identified changes in CER levels of patients treated with AAPs [48,49]. Schwarz and colleagues did not identify effects of AAPs on CER levels in post-mortem brain tissue samples [32]. However, Narayan and colleagues identified alterations in sphingolipid metabolism gene expression in post-mortem brain tissue of schizophrenia patients treated with typical antipsychotics compared to controls [50]. A study by Suvitaival and colleagues found associations between sphingolipid clusters and response to antipsychotics [51]. This study identified trend correlations between peripheral lipid and glucose measures as well. Additionally, relevant to our study in the skeletal muscle, Weston-Green and colleagues found no effect of 1-hour AAP treatment on CER muscle levels in female rats; however, they did identify decreases in hepatic CER levels [52]. Within our study, we identified elevated levels of skeletal muscle CERs in the AAP group relative to those in the mood stabilizer group, which may align with studies in the metabolic literature that have demonstrated increased skeletal muscle CERs with insulin resistance [53]. Our results may not align with that of the preclinical studies due to the populations being studied and the length of treatment with an AAP, amongst other factors.

### 3.2. Skeletal Muscle Lipids Correlate with AAP-Induced Insulin Resistance

Skeletal muscle lipid metabolism has been shown to play a role in insulin resistance by influencing the ability of the tissue to properly uptake glucose from the periphery [54,55,56,57]. Within our study, the AAP group had a higher rate of insulin resistance compared to the mood stabilizer group. Additionally, opposing relationships between insulin sensitivity and lipids were observed between the AAP and mood stabilizer groups. Qualitatively, the number of positive relationships for TFAs and PCs was greater in the AAP group while more negative relationships for CERs were observed in the AAP group when compared to those in the mood stabilizer group. Increased skeletal muscle diacylglycerol and CER levels have been linked to dysregulation of insulin signaling and insulin resistance, and it is hypothesized that CER may be particularly important in the pathogenesis of diabetes [58,59,60,61,62,63]. Our findings add to the potential importance of the CER class in medication-associated insulin resistance. Diacylglycerols can also be produced from the breakdown of PC by phospholipase D, and reduced skeletal muscle PC levels may be associated with insulin resistance in humans, suggesting a possible role for multiple lipids in insulin resistance [64,65]. Indeed, a study by Tonks and colleagues found increased levels of plasma and skeletal muscle sphingolipids (including CERs) and diacylglycerols and decreased lysoglycerolipids as being associated with adiposity-independent insulin resistance in humans [58]. For example, they identified an increase in skeletal muscle CER (18:1/18:0) in obese insulin-resistant subjects relative to obese insulin-sensitive subjects. In a similar manner, we identified an increase in CER (18:1/18:0) in insulin-resistant AAP-treated patients, relative to that in the more insulin-sensitive mood stabilizer-treated patients, which correlated with insulin sensitivity. Oresic and colleagues found that insulin-resistant schizophrenia patients on antipsychotics had lower peripheral shorter-chain lysoPC levels, such as 16:0, 18:0 and 20:3, compared to unaffected twins and healthy controls [29]. Additionally, within their study, they found that shorter-chain fatty acids, such as 14:0, 16:0 and 18:0, were both decreased in the serum of patients on AAPs but also correlated strongly with BMI and insulin resistance as measured by the Homeostatic Model Assessment of Insulin Resistance (HOMA-IR). Our study again supports these findings within the skeletal muscle where we identified lower levels of fatty acids of this length (i.e., 14:0, 16:0 and 18:0), which also correlated strongly with an ISI. Further prospective studies are needed, including work with antipsychotic-naïve patients, to understand the differing effects of antipsychotic-induced insulin resistance on tissue-specific lipidomes [66].

### 3.3. Limitations

Our work analyzed skeletal muscle lipid species within the classes of TFAs, PCs, and CERs. Although several significant relationships were observed, other areas of the lipidome, such as glycerolipids (e.g., diacylglycerols) and sphingolipids, require investigation to understand the effect of AAP treatment on the skeletal muscle lipidome. The analyses were conducted in a cross-sectional population which does not allow for causal conclusions to be made. Additionally, although we found no effect of sex on lipidomic profiles, this should be interpreted due to the larger amount of females in our sample and the overall limited number of included patients. Future work should be designed to analyze possible sex-specific differences in lipidomic features in humans as it relates to AAPs and skeletal muscle insulin resistance. Nevertheless, this is the first study to obtain skeletal muscle biopsies from a human population treated with AAPs and, as such, will serve as support for future prospective investigations into the effects of AAPs on the skeletal muscle lipidome. Prospective investigations will be critical for validating the findings here and potentially identifying biomarkers or treatment targets. Our comparisons were between a group of bipolar subjects on AAPs compared to a group on mood stabilizers and did not include a healthy control group, since the aim was to make comparisons based on medication status rather than psychiatric diagnosis. It has been demonstrated that medication-naïve patients with severe mental illness, as well as those receiving AAP treatment independent of psychiatric disease, have higher than average rates of insulin resistance and glucose dysregulation [45,67,68]. Our samples included patients on any AAP which allows for analysis of a class effect but not of specific AAPs. Therefore, our work does not rule out potential psychiatric effects on the skeletal muscle lipidomic features, nor does it allow for the potential individual mediation effects to be determined. Although all antipsychotics are associated with a degree of metabolic liability, certain AAPs are thought to carry greater risk than others [66,69]. To this end, the previous peripheral plasma or serum lipidomic studies of AAPs described above have indeed found potentially differing effects based on AAP types. Finally, our patients had BMI values in the obese range; therefore, the insulin resistance observed in our sample is likely at least in part due to being overweight/obese. Future work investigating the skeletal muscle lipidome and AAP-induced insulin resistance will need to account for obesity and weight gain.

## 4. Materials and Methods 

### 4.1. Subject Recruitment, Inclusion Criteria, and Clinical Assessment

All study protocol and procedures were reviewed and approved by the Wayne State University Institutional Review Board for human subject research (FWA 00002460 with approval through the M1-Medical adult IRB board IRB00000325). Potential participants were recruited from the Wayne State University and the surrounding area through public and clinic postings. Interested participants were invited to the Wayne State University Clinical Research Services Center and underwent fully informed consent and evaluated for the following inclusion criteria: (1) age 18–60 years, (2) a diagnosis of bipolar disorder I, II or NOS by the Mini International Neuropsychiatric Interview (MINI) [70], and (3) currently treated with AAP or mood stabilizer monotherapy at a consistent dose for at least 6 months and confirmed through pharmacy records (benzodiazepines and antidepressants were allowed but subjects could not be on both AAP and mood stabilizers concurrently). Potential participants were excluded if they (1) had a known or documented metabolic disease (e.g., prediabetes, diabetes, dyslipidemia, etc.) prior to beginning their AAP or mood stabilizer, (2) had a primary relative with a diabetes diagnosis, (3) were unable to undergo a muscle biopsy (e.g., allergy to lidocaine, history of bleeding, etc.), (4) were unable to obtain intravenous access for blood draws and muscle biopsy procedure, or (5) had an active diagnosis of substance abuse or dependence. 

For these participants meeting inclusion and exclusion criteria, they were enrolled in the study which consisted of a single visit to the clinical research services center after an overnight fast of at least 10 h. Participant height, weight and waist, and hip circumference were measured. An intravenous line was placed in the antecubital fossa for blood sampling. A 120-min oral glucose tolerance test was performed to calculate an ISI by the Matsuda method [39]. Insulin and glucose were analyzed by the Detroit Medical Center Laboratory (Detroit, MI, USA). 

### 4.2. Lipidomic Analysis of Biopsied Skeletal Muscle

#### 4.2.1. Lipidomic Classes Analyzed

At the beginning of the visit, participants underwent a fasting muscle biopsy in the *vastus lateralis* by a trained physician under local anesthesia using the modified Bergstrom needle technique [71]. The biopsied muscle was immediately cleaned of blood, connective tissue and fat and flash frozen. For lipidomic analysis, 20 mg of muscle was provided to the Wayne State University Lipidomic Core (Detroit, MI, USA) for analysis of 3 lipid classes: (1) fatty acyl TFAs (a total of esterified and non-esterified fatty acids) which we call a TFA panel, (2) a PC panel and (3) a CER panel. The core analyzed these three classes via their standardized methodology, which utilizes protocols from the Lipid Metabolites and Pathways Strategy (LIPID MAPS) consortium and the National Institute of Standards and Technologies to ensure comparability of analyses across laboratories and has been previously described [72,73,74,75]. 

#### 4.2.2. Total Fatty Acid Analysis

For the TFA analysis, total lipids in the sample were extracted by methyl tert-butyl ether according to published methods [76]. The extracted lipid was then dissolved in 1 mL methanol in a glass tube with a Teflon-lined cap, and aqueous sodium hydroxide was added to a final concentration of 1 M and the tube was flushed with nitrogen gas. The mixture was incubated at 37 °C for 2 h, followed by adjustment to a pH of 3–4 with dilute aqueous hydrochloric acid. The reaction mixture was diluted with water, such that the methanol concentration was less than 50%. Next, internal standards (5 ng each of myristic acid-d3, oleic acid-d17, stearic acid-d3, linoleic acid-d4, arachidonic acid-d8, and tetracosanoic acid-d4 from Avanti Polar Lipids, Alabaster, AL, USA) were added and then extracted with isooctane-ethyl acetate (9:1). The organic solvent extract was dried under nitrogen and reconstituted in methanol containing 0.1% ammonium acetate. The final extract was subjected to high-performance liquid chromatography–tandem mass spectrometry (HPLC–MS/MS) on a Prominence xR system (Shimadzu, Kyoto, Japan) coupled with a QTRAP5500 mass analyzer (SCIEX, Ontario, Canada). HPLC used a Persuit diphenyl column (Agilent, Santa Clara, CA, USA) with an eluent flow rate of 700 uL/min. Mobile phases A (water, 5 mM ammonium acetate and 2.1 mM acetic acid) ran at 45% for 2 min with 55% phase B (acetonitrile with 20% isopropanol), which increased linearly to 95% during 4 min. The coupled MS analysis followed in a negative electrospray ionization (ESI) mode with “pseudo-molecular” multiple reaction monitoring (MRM) methods published earlier [77].

#### 4.2.3. Phosphatidylcholine Analysis

To analyze PC lipid species profiles within each skeletal muscle sample, the samples were first extracted with methyl tert-butyl ether, as described above for the TFA analysis [76]. The extract was then fractionated by solid phase extraction (SPE) on an aminopropyl silica cartridge (Phenomenex, California, USA) according to the methods of Fauland and colleagues, and the PC fraction was collected [78]. The PC fraction was analyzed by the direct infusion and multiple precursor ion (*m*/*z* 184) scan method in the positive ion mode [79] using diheptanoyl phosphatidylcholine (Avanti Polar Lipids) as an internal standard on the QTRAP5500 mass analyzer.

#### 4.2.4. Ceramide Analysis

For the CER analysis, total sphingolipids along with mixed internal standards (C-17 analogs of the sphingolipids) were first extracted with isopropanol-ethyl acetate (3:7), as described by Bielawski and colleagues [80]. The extracted samples were analyzed for CERs using precursor ion scans (m/z 264). Sphingoid bases and their phosphates were analyzed by MRM methods following HPLC separation on C8 columns (HPLC–MS/MS) as follows: Briefly, HPLC was performed on a Prominence xR system using a Targa C8 (5 µ, 2.1 × 20 mm, Higgins Analytical) column. The mobile phase consisted of a gradient between A: methanol–water–ammonium formate–formic acid (5:95:1 mM:0.2, *v*/*v*) and B: methanol–ammonium formate–formic acid (100:2mM:0.2, *v*/*v*). The gradient program with respect to the composition of B was as follows: 0 min, 35%; 2 min, 100%; and 5 min, 100%. The flow rate was 0.5 mL/min. The HPLC eluate was directly introduced to an ESI source of QTRAP5500 mass analyzer (ABSCIEx) in the positive ion mode with following conditions: curtain gas: 35 psi, GS1: 45 psi, GS2: 45 psi, temperature: 650 °C, ion spray voltage: 5500 V, collision gas: low, declustering potential: 100 V, and entrance potential: 10 V. The eluate was monitored by the MRM method to detect unique molecular ion–daughter ion combinations for each of the sphingolipids and long chain bases (sphingosines) with 100 ms dwell time for each transition. Optimized collisional energies (16 to 28 eV) and collision cell exit potentials (10 V) were used for each MRM transition.

#### 4.2.5. Lipidomic Data Acquisition, Processing and Identification

The mass spectrometer data for each lipidomic analysis were acquired using Analyst 1.6.2 software (SCIEx), and the MRM transition chromatograms were quantitated by MultiQuant software 3.0.2 (SCIEx). The internal standard signal for each respective lipidomic analysis (i.e., 5 ng each of myristic acid-d3, oleic acid-d17, stearic acid-d3, linoleic acid-d4, arachidonic acid-d8, and tetracosanoic acid-d4 for TFAs, diheptanoyl phosphatidylcholine for PCs and C-17 analogs of sphingolipids for CERs) in each chromatogram was used for normalization for recovery as well as relative quantitation of each analyte. The MRM data were subjected to lipid identification by LipidView software (v1.2, SCIEx). Individual lipid species were indicated by either their common name for TFAs or by their fatty acid or lipid class and structure according to the experimental methods of lipid identification provided for each class above.

### 4.3. Statistical Analysis

The clinical data were presented in means ± standard deviation and compared between the AAP and mood stabilizer groups using either student’s *t*-test, chi-square test or Fisher exact test, where appropriate. For the lipidomic data, individual lipid values were kept for statistical analysis if they were detected in at least 75% of the samples. These lipids that were kept were tested for statistical normality by the Shapiro–Wilk test and all but one lipid species (Eicosanoic acid) did not meet the assumption of normality (*p* < 0.05). Non-normal lipid species were log-transformed which then met the assumption of normality by re-testing of Shapiro–Wilk test. An initial analysis was performed comparing the lipidomic profiles (independent *t*-tests) based on sex due to the potential importance of accounting for sex in lipidomic and metabolomics studies. Next, differences in lipid levels were compared between the AAP and mood stabilizer groups using independent, two-tailed *t*-tests. Fold change was calculated in the AAP group relative to the mood stabilizer group with raw fold changes above 1.5 and below 0.66 considered qualitatively important in our analyses and the log2 fold change was presented to provide an estimation of differences after log-transformation (in the data and figure forms). Pearson correlations were performed between lipids and insulin sensitivity to evaluate the potential interaction between these variables. Raw *p*-values were reported and multiple testing was corrected within each lipid class using the false discovery rate method, where only *q*-values of <0.05 were considered statistically significant along with our fold change cutoffs described above [81]. Statistical analyses were carried out with JMP 14 Pro Software (SAS, Cary, NC, USA).

## 5. Conclusions

Our study suggests that the skeletal muscle lipidome may be influenced by AAP treatment of psychiatric disease in humans. Furthermore, lipidomic associations with AAP treatment and insulin sensitivity may suggest that underlying changes in skeletal muscle lipid composition may play a role in AAP-associated insulin resistance. Future work utilizing prospective sampling with baseline measurements prior to treatment will be needed to confirm and expand on the findings here to identify potential targets for the treatment of AAP-associated metabolic side effects, with the goal of reducing cardiovascular morbidity and mortality in the severely mentally ill.

## Figures and Tables

**Table 1:** Sample set characteristics.

	Atypical Antipsychotic (*n* = 15)	Mood Stabilizer (*n* = 15)
Age (years)	43.0 ± 14.9	45.3 ± 13.0
Sex (% female)	61	57
Race (% Caucasian/% African-American)	57/34	71/28
WHR	1.00 ± 0.10	1.02 ± 0.04
BMI (mg/kg^2^)	32.0 ± 8.9	33.5 ± 6.3
Insulin Sensitivity Index *	5.20 ± 3.8	12.4 ± 6.90
Cholesterol (mg/dL)	173 ± 40.9	178 ± 38.9
LDL (mg/dL)	96.1 ± 34.1	103 ± 41.3
HDL (mg/dL)	57.4 ± 23.6	47.2 ± 15.8
Triglycerides (mg/dL)	95.3 ± 46.7	107 ± 53.8
Antipsychotic Type (%quetiapine/%risperidone/ %olanzapine/%lurasidone)	50/ 25/ 12.5/ 12.5	NA
Mood Stabilizer (%lamotrigine/%lithium/%valproic acid).	NA	40/33/27

Date presented as mean ± SD. or %. WHR: Waist-to-hip ratio. BMI: Body Mass Index. LDL: Low-density lipoprotein. HDL: High-density lipoprotein. * indicates *p*-value < 0.05.

**Table 2:** Lipidomic species analyzed.

Lipid	Raw Fold Change ^a^	Log2 Fold Change ^a^	Raw *p*-Value	FDR *q*-Value
***Total Fatty Acids***				
Lauric acid (C12:0)	0.41	−1.28	8.19 × 10^−5^	1.07 × 10^−3^ *
Myristic acid (C14:0)	0.59	−0.76	5.74 × 10^−3^	9.29 × 10^−3^ *
Pentadecylic acid (C15:0)	0.61	−0.70	2.24 × 10^−3^	5.72 × 10^−3^ *
Palmitic acid (C16:0)	0.50	−1.0	6.9 × 10^−3^	0.01 *
Palmitoleic acid (C16:1)	0.30	−1.72	0.02	0.03 *
Margaric acid (C17:0)	0.52	−0.96	3.32 × 10^−4^	1.56 × 10^−3^ *
Heptadecenoic acid (C17:1)	0.54	−0.89	0.18	0.22
Stearic acid (C18:0)	0.53	−0.92	1.24 × 10^−3^	4.5 × 10^−3^ *
Oleic acid (C18:1)	0.30	−1.7	0.02	0.03 *
Linoleic acid (C18:2)	0.37	−1.44	0.02	0.02 *
Arachidic acid (C20:0)	0.43	−1.21	7.66 × 19^−5^	1.07 × 10^−3^ *
Gondoic acid (C20:1)	0.10	−3.22	0.01	0.02 *
Eicosadienoic acid (C20:2)	0.17	−2.56	2.83 × 10^−3^	6.62 × 10^−3^ *
Dihomo-ү-linolenic acid (C20:3)	0.24	−2.08	3.88 × 10^−3^	8.23 × 10^−3^ *
Arachidonic acid (C20:4)	0.34	−1.56	1.93 × 10^−3^	5.49 × 10^−3^ *
Eicosapentaenoic acid (C20:5)	0.69	−0.53	0.88	0.92
Behenic acid (C22:0)	0.48	−1.06	3.72 × 10^−4^	1.56 × 10^−3^ *
Erucic acid (C22:1)	0.24	−2.07	9.01 × 10^−4^	3.92 × 10^−3^ *
Docosadienoic acid (C22:2)	0.16	−2.64	1.94 × 10^−3^	5.49 × 10^−3^ *
Docosatrienoic acid (C22:3)	0.69	−0.53	0.96	0.96
Adrenic Acid (C22:4)	0.60	−0.73	0.23	0.28
Osbond acid (C22:5)	0.60	−0.73	0.32	0.36
Docosahexaenoic acid (C22:6)	0.39	−1.37	0.60	0.65
Lignoceric acid (C24:0)	0.46	−1.12	4.86 × 10^−3^	9.29 × 10^−3^ *
Nervonic acid (C24:1)	0.35	−1.51	2.98 × 10^−4^	1.56 × 10^−3^ *
Cerotic acid (C26:0)	0.46	−1.12	0.02	0.02*
***Phosphatidylcholines***				
PC 32:0	0.55	−0.86	0.01	0.04 *
PC 32:1	0.69	−0.54	0.09	0.12
PC 34:1	0.54	−0.88	0.02	0.04 *
PC 34:2	0.62	−0.70	0.04	0.05
PC 34:3	0.50	−1.0	4.01 × 10^−3^	0.04 *
PC 36:1	0.61	−0.71	0.01	0.04 *
PC 36:2	0.67	−0.57	0.53	0.58
PC 36:3	0.59	−0.76	0.03	0.04 *
PC 36:4	0.58	−0.80	0.02	0.04 *
PC 38:4	0.61	−0.72	0.04	0.05
PC 38:5	0.86	−0.22	0.80	0.80
***Ceramides***				
CER(d18:1/14:0)	4.00	2.00	1.34 × 10^−3^	5.21 × 10^−3^ *
CER(d18:1/16:0)	4.29	2.10	1.49 × 10^−3^	5.21 × 10^−3^ *
CER(d18:0/16:0)	6.21	2.63	3.32 × 10^−4^	4.65 × 10^−3^ *
CER(d18:1/18:0)	3.50	1.81	0.01	0.01 *
CER(d18:0/18:0)	4.55	2.19	0.02	0.02 *
CER(d18:1/18:1)	3.61	1.85	1.17 × 10^−3^	5.21 × 10^−3^ *
CER(d18:1/18:2)	2.91	1.54	2.34 × 10^−3^	6.56 × 10^−3^ *
CER(d18:1/20:0)	4.03	2.01	6.51 × 10^−3^	0.01 *
CER(d18:1/24:0)	2.54	1.34	0.02	0.02 *
CER(d18:0/24:0)	2.34	1.23	0.02	0.02 *
CER(d18:1/24:1)	3.76	1.91	3.29 × 10^−3^	7.68 × 10^−3^ *
CER(d18:1/26:0)	2.43	1.28	7.26 × 10^−3^	0.01 *
CER(d18:0/26:0)	2.86	1.52	4.07 × 10^−3^	8.15 × 10^−3^ *
CER(d18:1/26:1)	2.44	1.29	0.05	0.05

Table 2 shows individual lipid species included in analyses. Lipids are from the main classes of total fatty acids (TFAs), phosphatidylcholines (PCs) and ceramides (CERs) and are depicted by their common names. The fold change, raw *p*-value and false discovery rate-corrected *q*-value are shown. A raw fold change of >1.5 or <0.66 was considered qualitatively important. * indicates a statistically significant difference between the atypical antipsychotic (AAP) and mood stabilizer groups at a *q*-value < 0.05. ^a^ Fold change (raw or log) is the mean AAP level relative to the mean mood stabilizer level.

## Supplementary Materials

Supplementary materials can be found at http://www.mdpi.com/2218-1989/8/4/64/s1. Table S1: Comparison of lipidomic profiles based on sex, Figure S1: *p*-value heatmap of insulin sensitivity and TFAs. The heatmap shows degree of significance between individual TFA species and insulin sensitivity measured by the Matsuda ISI. Lipids are ordered by the average ascending *p*-value. AAP: atypical antipsychotic, and MS: mood stabilizer, Figure S2: *p*-value heatmap of insulin sensitivity and PCs. Heatmap shows degree of significance between individual PC species and insulin sensitivity measured by the Matsuda ISI. Lipids are ordered by the average ascending *p*-value. AAP: atypical antipsychotic, and MS: mood stabilizer, Figure S3. *p*-value heatmap of insulin sensitivity and CERs. The heatmap shows degree of significance between individual CER species and insulin sensitivity measured by the Matsuda ISI. Lipids are ordered by the average ascending *p*-value. AAP: atypical antipsychotic, and MS: mood stabilizer. The raw lipidomic data will be made available to the public through our institutions “Data@Wayne” WSU Health Data Catalog, which can be accessed through the following link: https://guides.lib.wayne.edu/data_at_wayne.

## Abbreviations

MDPI	Multidisciplinary Digital Publishing Institute
DOAJ	Directory of Open Access Journals
TLA	three-letter acronym
LD	linear dichroism
AAP	atypical antipsychotic
TFA	total fatty acid
PC	phosphatidylcholine
CER	ceramide
