# Supplementary material for: Atypical Antipsychotics and the Human Skeletal Muscle Lipidome
Source: Metabolites. 2018 Oct 13;8(4):64. doi: 10.3390/metabo8040064 (PMC6316471; doi:10.3390/metabo8040064)
Supplement: Supplementary file 1 [file metabolites-08-00064-s001.pdf]

Supplementary Information

Supplementary Table S1. Comparison of Lipidomic Profiles based on Sex.

Lipid level	Log2 Fold Change	Raw P-value
Lauric acid (C12:0)	-0.43938639	0.23
Myristic acid (C14:0)	-0.27951771	0.63
Pentadecylic acid (C15:0)	-0.35873748	0.18
Palmitic acid (C16:0)	-0.76701498	0.08
Palmitoleic acid (C16:1)	1.35180842	0.51
Margaric acid (C17:0)	-0.35383418	0.20
Heptadecenoic acid (C17:1)	0.116191787	0.87
Stearic acid (C18:0)	-0.63055655	0.08
Oleic acid (C18:1)	0.738683006	0.55
Linoleic acid (C18:2)	0.572218845	0.96
Arachidic acid (C20:0)	-0.56936565	0.10
Gondoic acid (20:1)	0.48534323	0.67
Eicosadienoic acid (20:2)	0.676759009	0.91
Dihomo-γ-linolenic acid (C20:3)	0.35506948	0.68
Arachidonic acid (C20:4)	-0.06193689	0.69
Eicosapentaenoic acid (C20:5)	0.034324104	0.35
Behenic acid (C22:0)	-0.62780661	0.05
Erucic acid (C22:1)	-0.18602999	0.47
Docosadienoic acid (C22:2)	0.295363406	0.77
Docosatrienoic acid (C22:3)	0.508885606	0.62
Adrenic Acid (C22:4)	0.211796723	0.93
Osbond acid (22:5)	-0.09139106	0.96
Docosahexaenoic acid (22:6)	-1.18078349	0.90
Lignoceric acid (C24:0)	-0.72592143	0.06
Nervonic acid (C24:1)	-0.51559304	0.17
Cerotic acid (C26:0)	-0.79832112	0.10
PC 32:0	0.010315881	0.64
PC 32:1	0.138561078	0.61
PC 34:1	-0.17804042	0.93
PC 34:2	-0.25285831	0.65
PC 34:3	-0.31971286	0.32
PC 36:1	0.149911987	0.68
PC 36:2	-0.29553172	0.46
PC 36:3	-0.06305537	0.83

PC 36:4	-0.11428972	0.95
PC 38:4	-0.14717918	0.80
PC 38:5	-0.12312788	0.88
CER(d18:1/14:0)	-0.20352713	0.99
CER(d18:1/16:0)	0.327480498	0.83
CER(d18:0/16:0)	-0.39263563	0.83
CER(d18:1/18:0)	-0.37999395	0.59
CER(d18:0/18:0)	-0.47640719	0.55
CER(d18:1/18:1)	-0.19178349	0.65
CER(d18:1/18:2)	0.00772969	0.58
CER(d18:1/20:0)	0.062930433	0.56
CER(d18:1/24:0)	0.202657777	0.29
CER(d18:0/24:0)	-0.02026895	0.61
CER(d18:1/24:1)	0.12772296	0.66
CER(d18:1/26:0)	0.067850969	0.38
CER(d18:0/26:0)	0.321054188	0.24
CER(d18:1/26:1)	-0.20086263	0.92

Table shows individual lipid species included in analyses. Lipids are from the main classes of Total Fatty Acids (TFAs), Phosphatidylcholines (PCs) and Ceramides (CERs) and are depicted by their common name. Mean log2 fold change of male relative to female sex. Unadjusted P-values for comparison between sex using an independent t-test on log-transformed values are shown.
*** indicates a statistically significant difference between the Atypical Antipsychotic (AAP) and mood stabilizer group at a q-value<0.05.**

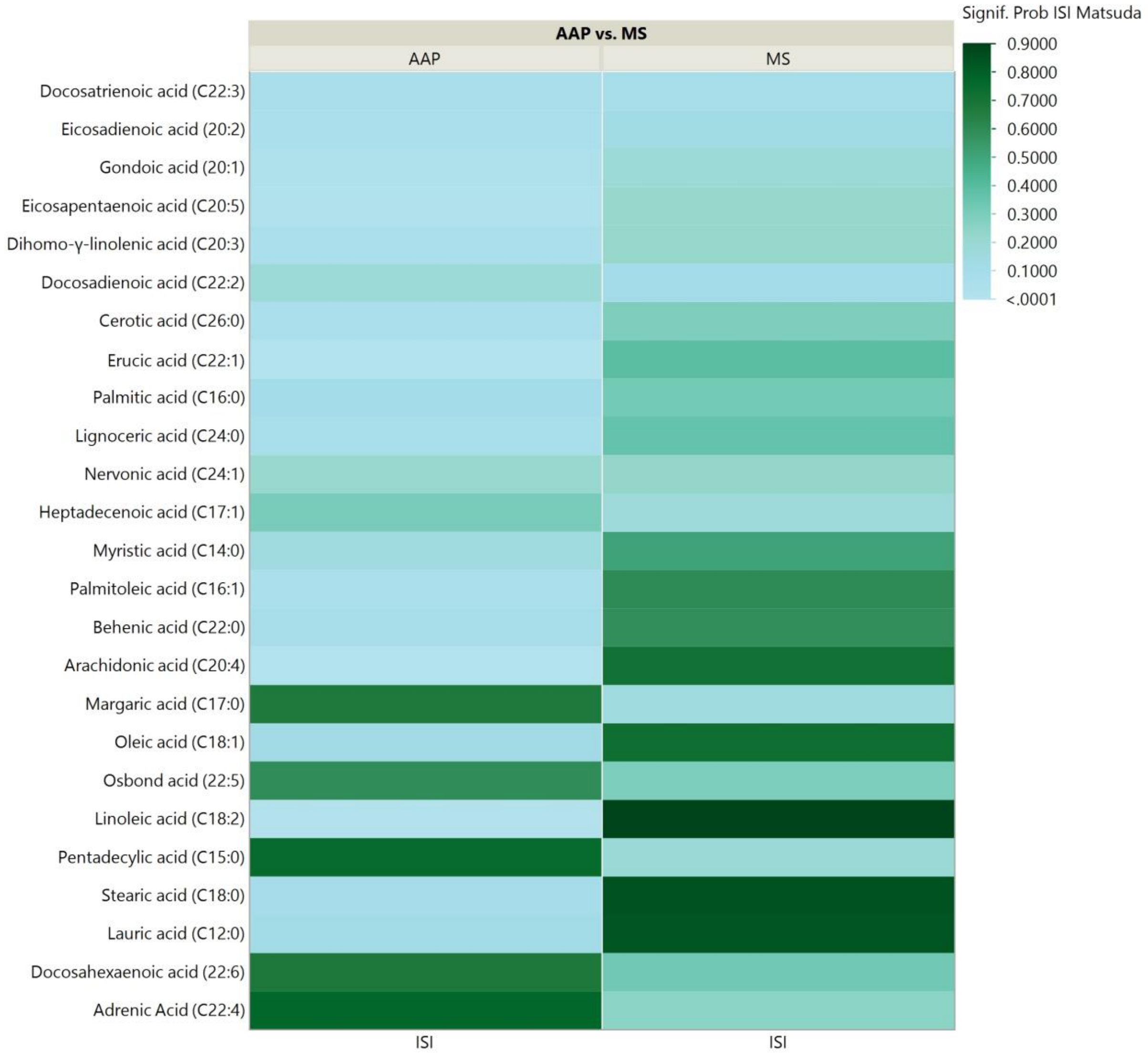

Supplementary Figure S1. P-value Heatmap of Insulin Sensitivity and Total Fatty Acids. Heatmap shows degree of significance between individual total fatty acid species and insulin sensitivity measured by the Matsuda Insulin Sensitivity Index (ISI). Lipids are ordered by average ascending p-value. AAP: atypical antipsychotic, MS: mood stabilizer

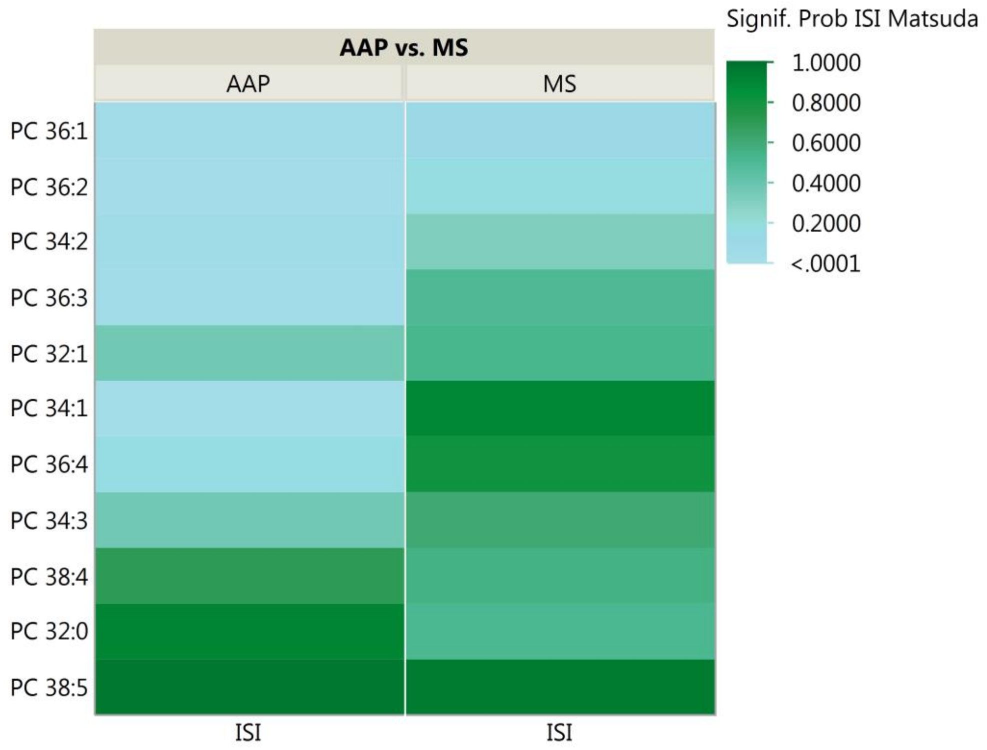

Supplementary Figure S2. P-value Heatmap of Insulin Sensitivity and Phosphatidylcholines. Heatmap shows degree of significance between individual phosphatidylcholines species and insulin sensitivity measured by the Matsuda Insulin Sensitivity Index (ISI). Lipids are ordered by average ascending p-value. AAP: atypical antipsychotic, MS: mood stabilizer

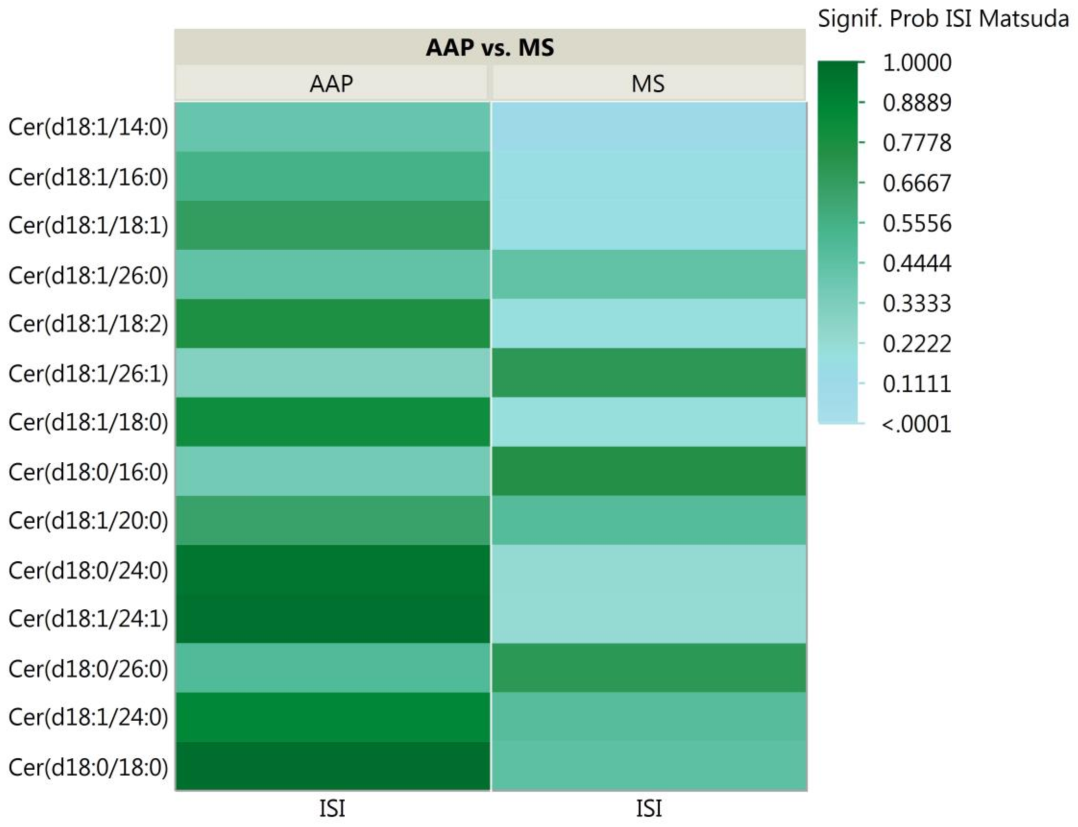

Supplementary Figure S3. P-value Heatmap of Insulin Sensitivity and Ceramides. Heatmap shows degree of significance between individual ceramide species and insulin sensitivity measured by the Matsuda Insulin Sensitivity Index (ISI). Lipids are ordered by average ascending p-value. AAP: atypical antipsychotic, MS: mood stabilizer
